# Supplementary material for: Analysis of multi-condition single-cell data with latent embedding multivariate regression
Source: Nat Genet. 2025 Jan 3;57(3):659–67. doi: 10.1038/s41588-024-01996-0 (PMC11906359; doi:10.1038/s41588-024-01996-0)
Supplement: Supplementary file 1 — Supplementary Note 1 [file 41588_2024_1996_MOESM1_ESM.pdf]

nature genetics

Supplementary information https://doi.org/10.1038/s41588-024-01996-0

Analysis of multi-condition single-cell data with latent embedding multivariate regression

In the format provided by the authors and unedited

Contents

Implementation 1
Non-distance preserving extension 1
Post-processing 1
Cluster-free differential expression 2
Differential expression neighborhoods 3
Pseudobulk differential expression analysis 3
Relation to other methods 4
Relation to GSFA 4
Relation to GSFA 4
Relation to GEDI 4
Relation with interaction models 4

Implementation

We fit multi-condition PCA, Eqn. (4) by:

1. Solving the linear regression for $\mathbf{\Gamma}$, treating $\mathbf{R}(x)$ and $\mathbf{Z}$ as 0.

2. Optimizing on the Grassmann manifold for the parameters $\mathbf{B}$ of the function $\mathbf{R}$, keeping Γ fixed.

3. Inferring $\mathbf{Z}_{:c}$ by projecting $\mathbf{Y}_{:c}$ on the orthonormal basis $\mathbf{R}(\mathbf{X}_{:c})$.

In Step 1, we solve a linear regression for $\mathbf{\Gamma}$

$$\hat{\mathbf{\Gamma}} = \arg\min_{\mathbf{\Gamma}} \|\mathbf{Y} - \mathbf{\Gamma}\mathbf{X}^T\|_2^2. \tag{11}$$

In Step 2, we need to solve the manifold regression problem

$$\arg\min_{\mathbf{B}_{::k} \in \mathcal{T}_{\mathbf{o}}\mathrm{Gr}(G,P)} \left| \tilde{\mathbf{Y}}_{:c} - \mathrm{Exp}_{\mathbf{o}}\left(\sum_k \mathbf{B}_{::k}\mathbf{X}_{ck}\right)\mathbf{Z}\right|^2, \tag{12}$$

where $\tilde{\mathbf{Y}} = \mathbf{Y} - \hat{\mathbf{\Gamma}}\mathbf{X}^T$. We choose the base point $\mathbf{o}$ as the first P principal vectors of fitting PCA to full, centered data matrix $\tilde{\mathbf{Y}}$.

To optimize $\mathbf{B}$, we build on the work of Kim et al.[1]. They developed an algorithm to approximate the geodesic regression problem

$$\arg\min_{\mathbf{B}_{::k} \in \mathcal{T}_{\mathbf{o}}\mathcal{M}} d\left(\Omega_i, \mathrm{Exp}_{\mathbf{o}}\left(\sum_k \mathbf{B}_{::k}\mathbf{X}_{ik}\right)\right), \tag{13}$$

where $\mathcal{M}$ is a Riemannian manifold, $\Omega_i \in \mathcal{M}$ are data points on the manifolds, and

$$d(p,q) = \sqrt{\langle \mathrm{Log}(p,q), \mathrm{Log}(p,q)\rangle} \tag{14}$$

is the geodesic distance between two points on $\mathcal{M}$. Here, Log is the inverse of the exponential map.

If the observations Ω_i are close to each other, the solution to Eqn. (13) is well approximated by the solution to a standard linear regression in the tangent space

$$\arg\min_{\mathbf{B}_{::k} \in \mathcal{T}_{\mathbf{o}}\mathcal{M}} \left(\mathrm{Log}(\mathbf{o}, \Omega_i) - \left(\textstyle\sum_k \mathbf{B}_{::k}\mathbf{X}_{ik}\right)\right)^2 \tag{15}$$

for a base point $o \in \mathcal{M}$ that is close to the center of all Ω_i.

Another step is required before we can apply Kim et al.[1]'s algorithm, since in our case, the observations $\tilde{\mathbf{Y}}_{:c}$ are not elements of the manifold (in our case, $\mathcal{M}$ is the Grassmann manifold $\mathrm{Gr}(G,P)$). We partition $\{1,\ldots,C\}$, the set of all cells, into sets of cells that share the same condition: $\mathbb{D}_1 \cup \ldots \cup \mathbb{D}_D = \{1,\ldots,C\}$ and $\forall d \in 1,\ldots,D : \forall c_1, c_2 \in \mathbb{D}_d : \mathbf{X}_{c_1:} = \mathbf{X}_{c_2:}$. Then, for each d (i.e., for each group of cells under the same conditions) we find an orthonormal basis $\mathbf{U}_d \in \mathrm{Gr}(G,P)$ using PCA on $\tilde{\mathbf{Y}}_{:\mathbb{D}_d}$, the data for these cells only. We then approximate a solution of Eqn. (12) by linear regression weighted by the number of observations per condition ($\#\mathbb{D}_d$) on the U_d projected into the tangent space of $\mathbf{o}$. For this, we plug $\Omega_i = \mathbf{U}_d$ into Eqn. (15) with

$$\mathrm{Log}^{(\mathrm{Gr})}(\mathbf{A},\mathbf{B}) = \mathbf{U}\,\mathrm{diag}(\tan^{-1}(d))\,\mathbf{V}^T, \tag{16}$$

where $\mathbf{U}$, $\mathbf{V}$, and d come from an SVD of $(\mathbf{B} - \mathbf{A}\mathbf{A}^T\mathbf{B})(\mathbf{A}^T\mathbf{B})^{-1} = \mathbf{U}\,\mathrm{diag}(d)\mathbf{V}^T$[2].

Non-distance preserving extension

After fitting the model described in the previous section, the user can choose to also fit a non-distance preserving extensions. The non-distance preserving term $\mathbf{S}$ is defined by the the parameters of $\mathbf{W}$ and $\mathbf{W}^{(0)}$, which we fit using ridge regression with a user-specified penalty that defaults to $\lambda = 0.01$.

Post-processing

After fitting the LEMUR model, we adjust the base space so that the rows of $\mathbf{Z}$ are sorted in descending order of their variance, i.e., we take our specific set of basis vectors and adjust them so that they may be interpreted analogously to principal components, as pointing in the direction of highest, second-highest, ... variance. Specifically, we calculate a singular value decomposition of $\mathbf{Z}$

$$\mathbf{Z} = \mathbf{U}\,\mathrm{diag}(d)\mathbf{V}^T. \tag{17}$$

Table 1 | Notation used in the manuscript.

Symbol	Meaning
$\mathbf{Y}'$	Raw count data ($\mathbf{Y}' \in \mathbb{Z}_{\geq 0}^{G\times C}$)
$\mathbf{Y}$	Data after size factor normalization and variance-stabilizing transformation ($\mathbf{Y} \in \mathbb{R}^{G\times C}$)
$\tilde{\mathbf{Y}}$	Data after regressing out the linear effects ($\mathbf{Y} - \gamma(\mathbf{X})$)
$\mathbf{X}$	Design matrix ($\mathbf{X} \in \mathbb{R}^{C\times K}$)
$\mathbf{Z}$	Position of each cell in the low-dimensional embedding ($\mathbf{Z} \in \mathbb{R}^{P\times C}$)
$\mathbf{\Delta}$	Predicted differential expression values between two conditions ($\mathbf{\Delta} \in \mathbb{R}^{G\times C}$)
P	Number of embedding dimensions. The associated index variable is p.
K	Number of covariates. The index variable is k.
G	Number of genes. The index variable is g.
C	Number of cells. The index variable is c.
$\gamma(x)$	Function that takes a vector of covariates (a row of $\mathbf{X}$) and returns a vector with the offset for each gene ($\gamma(x) \in \mathbb{R}^{G}$)
$\mathbf{\Gamma}$	Linear coefficients of $\gamma(x)$ ($\mathbf{\Gamma} \in \mathbb{R}^{G\times K}$)
$\mathbf{R}(x)$	Function that takes a vector of covariates (a row of $\mathbf{X}$) and returns a matrix with orthonormal columns ($\mathbf{R}(x) \in \mathrm{Gr}(G, P)$). Its role is analogous to the principal vectors in PCA.
$\mathbf{B}$	3-tensor of parameters that determines $\mathbf{R}(x)$. Each slice $\mathbf{B}_{::k}$ represents an element of the tangent space $\mathcal{T}_{\mathbf{o}}\,\mathrm{Gr}(G, P)$.
$\mathbf{o}$	The base-point ("zero") for the Grassmann exponential map ($\mathbf{o} \in \mathrm{Gr}(G, P)$)
$\mathbf{S}(x)$	Function that takes a vector of covariates and returns an invertible matrix ($\mathbf{S}(x) \in \mathbb{R}^{P\times P}$)
$\mathbf{W}$	3-tensor of parameters for $\mathbf{S}(x)$. Each slice $\mathbf{W}_{::k} \in \mathbb{R}^{P\times P}$

We then set the base point to

$$\tilde{\mathbf{o}} = \mathbf{o}\,\mathbf{U}, \tag{18}$$

adjust the coefficients of $\mathbf{R}$ to

$$\tilde{\mathbf{B}}_{::k} = \mathbf{B}_{::k}\,\mathbf{U} \tag{19}$$

and set the low-dimensional embedding $\mathbf{Z}$ to

$$\tilde{\mathbf{Z}} = \mathrm{diag}(d)\mathbf{V}^T. \tag{20}$$

Cluster-free differential expression

The parametric model of the multi-condition single-cell data learned by LEMUR can be used for intra- and extrapolation, that is, for prediction. First, for any observed cell, it predicts its gene expression in any of the conditions, even though each cell was only observed exactly once. Second, as each cell is parameterized by a position in latent space, the model can also predict expression of "synthetic cells" at any (unobserved) position z in the latent space for any condition x. We use these capabilities for differential expression analysis. Using the inferred parameters for $\gamma(x)$, $\mathbf{R}(x)$, and $\mathbf{S}(x)$, we write

$$f(x, z) = \mathbf{R}(x)\mathbf{S}(x)z + \gamma(x), \tag{21}$$

where f is a function that predicts the gene expression of a cell at latent space position z in the embedding space for any condition x.

Thus, the predicted differential expression for all genes in cell c between conditions 1 and 2

($d_1, d_2 \in \mathbb{R}^K$) is

$$\mathbf{\Delta}_{:c} = f(d_2, \mathbf{Z}_{:c}) - f(d_1, \mathbf{Z}_{:c}). \tag{22}$$

Differential expression neighborhoods

We use a stochastic sampling algorithm that works on the differential expression matrix $\mathbf{\Delta}$ to identify *neighborhoods* of cells that show consistent differential expression. Intuitively, these are intended to be sets of cells that cluster together in latent space, i.e., are similar or related cell types and cell states, that also show consistent differential expression with respect to the contrast of interest.

In a first step, we sample many one-dimensional projections of the embedding $\mathbf{Z}$. Specifically, we repeat the following N times: randomly sample two cells from $\{1, \ldots, C\}$, compute the vector between them, $v = \mathbf{Z}_{:c_1} - \mathbf{Z}_{:c_2}$, and project the data from all cells onto v. This results in the matrix (w_{cn}), where $n = 1, \ldots, N$ indexes the random samples and c indexes the cells. We choose N large enough that there is a good chance that interesting differential expression patterns are apparent in one or more of the $w_{:n}$'s.

Next, for each gene g, we identify the best of these one-dimensional data projections by choosing an n_g for which $w_{:n_g}$ has the maximal absolute correlation to $\mathbf{\Delta}_{g:}$. Intuitively, this selects a direction in latent space along which there is differential expression for that gene. We compute the order statistic $(i_1, \ldots, i_C)$ of $w_{:n_g}$, for each $c = 1, \ldots, C$ compute the t-statistic for the sample $\mathbf{\Delta}_{g,i_1}, \ldots, \mathbf{\Delta}_{g,i_c}$, choose the c_g which maximizes that statistic, and set $\mathbb{Q}_g = \left\{i_1, \ldots, i_{c_g}\right\}$.

Pseudobulk differential expression analysis

The pseudobulk approach accounts for the fact that the most relevant unit of replication in multi-condition single-cell data is the sample (and not the cells)[3]. It works by summing the counts across cells within a sample from the same cell subpopulation (e.g., "cell type"). If the data were obtained from F samples, the information which cell belongs to which sample implies a partition of $\{1, \ldots, C\}$ into F sets of cells, which we call $\mathbb{F}_1, \ldots, \mathbb{F}_F$.

Let $\mathbf{Y}' \in \mathbb{Z}_{\geq 0}^{G \times C}$ be the count matrix from which $\mathbf{Y}$ was constructed. Then we form the pseudobulk count matrix $\mathbf{V} \in \mathbb{Z}^{G \times F}$ as

$$\mathbf{V}_{gf} = \sum_{c \in \mathbb{F}_f \cap \mathbb{Q}_g} \mathbf{Y}'_{gc}, \tag{23}$$

for $f = 1, \ldots, F$, and calculate a gene-specific size factor

$$\mathrm{sf}_{gf} = \sum_{c \in \mathbb{F}_f \cap \mathbb{Q}_g} \sum_{h=1}^{G} \mathbf{Y}'_{hc}. \tag{24}$$

This construction differs from the usual pseudobulk approach as it uses a different set of cells $\mathbb{Q}_g$ for each gene.

To address the post-selection inference problem (we want to make inference on the statistical significance of an observed trend based on a test statistic that was itself constructed after consulting the data), we compute (23) on a set of held out cells, which are matched via the (w_{cn}) matrix. The fraction of cells that we assign to the training set (which is used to compute the neighborhoods $\mathbb{Q}_1, \ldots, \mathbb{Q}_G$), and to the test set (which is used to compute the pseudobulk data (23)) balances the power for accurately identifying neighborhoods with interesting gene expression changes, versus the power to provide statistical significance statements for those identifications. Future work may find more elegant solutions.

Relation to other methods

Relation to PCA regression and partial least squares regression

For linear regression problems

$$\mathbf{Y} = \mathbf{B}\mathbf{X}^T, \tag{25}$$

where the design matrix contains many (and potentially some colinear) columns, PCA regression replaces the original design matrix with a lower dimensional approximation $\mathbf{V}$ produced with PCA:

$$\mathbf{Y} = \mathbf{B}'\mathbf{V}^T. \tag{26}$$

Partial least squares is used in similar circumstances but chooses the lower dimensional approximation $\mathbf{V}$ such that it is optimal for predicting $\mathbf{Y}$[4].

The difference to the LEMUR model is that partial least squares and principal component regression find approximations of the design matrix, whereas LEMUR finds low-dimensional subspaces that approximate $\mathbf{Y}$ and expresses the relation of the subspaces as regression problem.

Relation to GSFA

Zhou et al.[5] described a model for the analysis of perturbation single-cell data called *guided sparse factor analysis* (GSFA) which is related to the SupSVD model[6]. It is built around matrix factorization of the observed data

$$\begin{aligned} \mathbf{Y} &\approx \mathbf{R}\mathbf{Z} \\ \mathbf{Z} &\approx \boldsymbol{\beta}\mathbf{X}^T. \end{aligned} \tag{27}$$

The model uses sparse priors on $\mathbf{R}$ and $\boldsymbol{\beta}$ to identify which gene modules are related to which latent factors. Unlike LEMUR, model (27) makes the latent embedding Z dependent on the design matrix $\mathbf{X}$.

The sparsity priors favor situations where a perturbation only affects a small number of latent factors in $\mathbf{Z}$ and thus columns of $\mathbf{R}$. In that case, the contrast between the subspaces spanned by the active factors of $\mathbf{R}$ and the inactive factors for a perturbation has a similar interpretation as the corresponding comparison in LEMUR. However, LEMUR directly identifies the subspaces per condition and does not need to rely on the indirect effect of the sparsity priors.

Relation to GEDI

A recent preprint by Madrigal et al.[7], published after the first preprint of this work (doi: 10.1101/2023.03.06.531268), presents a model for cluster-free differential expression analysis, similar to what we delineate by Step 1 in Fig. 1A. Their model can be summarized as

$$\mathbf{Y}_{:\mathbb{F}_f} = \mathbf{R}(\mathbf{X}_{f:})\mathbf{Z}_{:\mathbb{F}_f} + \boldsymbol{\Gamma}\mathbf{X}_{f:}^T, \tag{28}$$

where $\mathbb{F}_f$ are the indices of all cells from sample f and $\mathbf{X}$ is a design matrix on the sample level ($F \times K$). They define the function $\mathbf{R}$ as the linear combination of a reference state (θ_r) and deviations from that reference state (δ)

$$\mathbf{R}(x) = \theta_r + \sum_k x_k \delta_k. \tag{29}$$

This model is fairly similar to LEMUR as it also works by treating the subspace as a function of the covariates. The biggest difference with our model is that their $\mathbf{R}$ does not enforce the orthonormality constraints on $\mathbf{R}(x)$. The resulting degeneracy between $\mathbf{R}$ and $\mathbf{Z}$ is partially resolved by scaling each row of $\mathbf{Z}$ to a length of 1 and regularization on $\mathbf{R}$, which can optionally be guided by gene-regulatory networks.

Relation to interaction models

Model (4) can express interactions between known covariates and the latent position of each cell. For example, a drug perturbation might affect the gene expression of cells early in a developmental trajectory more than in mature cells. Our model simultaneously identifies the latent position and the interacting drug effect. Yet, the way the interactions are modeled here differs from that in ordinary linear models.

Interactions in ordinary linear models are formed using a direct (Hadamard) product between two or more known covariates. For example, the effectiveness of trastuzumab on breast cancer cells depends on their HER2 status, i.e., the drug is more effective if the HER2 protein level is high. Accordingly, we could model cell viability as a function of

$$\hat{y} = \beta_0 + \beta_1\, x_{\text{conc.}} + \beta_2\, x_{\text{HER2}} + \beta_3\, x_{\text{conc.}} \odot x_{\text{HER2}} \tag{30}$$

and call β_3 the interaction coefficient.

The LEMUR multi-condition PCA model (4) and the interaction model (30) are closely related.

To demonstrate, we define $\mathbf{R}$ not as eq. (5) but as

$$\mathbf{R}(x) = \sum_k \mathbf{B}_{::k} x_k, \tag{31}$$

and assume that $\mathbf{X}$ contains the known drug concentration from our example above, while $\mathbf{Z}$ contains the latent *HER2* status:

$$\mathbf{X} = \begin{pmatrix} | & | \\ 1 & x_{\text{conc.}} \\ | & | \end{pmatrix}, \; \mathbf{Z} \; = \begin{pmatrix} | & | \\ 1 & x_{\text{HER2}} \\ | & | \end{pmatrix}^T. \tag{32}$$

When we plug eqn. (31) into $\hat{\mathbf{Y}}_{:c} = \mathbf{R}(\mathbf{X}_{c:})\mathbf{Z}_{c:}$, we can rewrite it as

$$\hat{\mathbf{Y}} = \sum_{p,k} \mathbf{B}_{:kp} \left(\mathbf{X}_{:k} \odot \mathbf{Z}_{p:}\right), \tag{33}$$

which is just a different way to write eq. (30). This demonstrates that the only difference between the interaction model with latent factors and LEMUR is the choice of $\mathbf{R}$.

Interaction model (33) has been used to model the effects of regulatory variants in single-cells across cell states[8,9]. There, the cell states were represented using continuous factors $\mathbf{Z}$; however, the estimation proceeded step-wise: first, estimating $\mathbf{Z}$ using PCA or Harmony and only then fitting the interaction coefficients $\mathbf{B}$.

Independent of the parametrization (Eqn. (5) or Eqn. (31)), $\mathbf{R}(x)$ can be interpreted as spanning the space that best approximates the observations from condition x. The advantage of Eqn. (5) is that the constraints of the Grassmann manifold naturally map to this intuition. In contrast, the parametrization of Eqn. (31) does not enforce orthonormality between the columns of $\mathbf{R}(x)$, it does not even enforce a common scale. This makes the model degenerate when inferring $\mathbf{Z}$ and $\mathbf{B}$ simultaneously.

Geometrically, the columns of $\mathbf{B}$ in Eqn. (31) that correspond to the intercept in $\mathbf{X}$ span a base space. All other columns in $\mathbf{B}$ are vectors that point out of that base space. In contrast, the $\mathbf{B}_{::k} \in \mathcal{T}_{\mathbf{o}}\mathrm{Gr}(G, P)$ in Eqn. (5) correspond to rotations of the base space. For small angles between the spaces of two conditions, there is little difference between a rotation and the straight vector. Thus, one can interpret our multi-condition PCA model as approximating a conventional interaction model between observed and latent covariates.

Supplementary references

[1] Hyunwoo J Kim, Nagesh Adluru, Maxwell D Collins, Moo K Chung, Barbara B Bendlin, Sterling C Johnson, Richard J Davidson, and Vikas Singh. Multivariate general linear models (MGLM) on Riemannian manifolds with applications to statistical analysis of diffusion weighted images. In *Proceedings of the IEEE Conference on Computer Vision and Pattern Recognition*, pages 2705–2712, 2014. doi: 10.1109/CVPR.2014.352.

[2] P-A Absil, Robert Mahony, and Rodolphe Sepulchre. Riemannian geometry of Grassmann manifolds with a view on algorithmic computation. *Acta Applicandae Mathematica*, 80:199–220, 2004. doi: 10.1023/B:ACAP.0000013855.14971.91.

[3] Helena L Crowell, Charlotte Soneson, Pierre-Luc Germain, Daniela Calini, Ludovic Collin, Catarina Raposo, Dheeraj Malhotra, and Mark D Robinson. Muscat detects subpopulation-specific state transitions from multi-sample multi-condition single-cell transcriptomics data. *Nature Communications*, 11(1):6077, 2020. doi: 10.1038/s41467-020-19894-4.

[4] Svante Wold, Michael Sjöström, and Lennart Eriksson. PLS-regression: a basic tool of chemometrics. *Chemometrics and Intelligent Laboratory Systems*, 58(2):109–130, 2001. doi: https://doi.org/10.1016/S0169-7439(01)00155-1.

[5] Yifan Zhou, Kaixuan Luo, Lifan Liang, Mengjie Chen, and Xin He. A new Bayesian factor analysis method improves detection of genes and biological processes affected by perturbations in single-cell CRISPR screening. *Nature Methods*, 20:1693–1703, 2023. doi: 10.1038/s41592-023-02017-4.

[6] Gen Li, Dan Yang, Andrew B Nobel, and Haipeng Shen. Supervised singular value decomposition and its asymptotic properties. *Journal of Multivariate Analysis*, 146:7–17, 2016. doi: 10.1016/j.jmva.2015.02.016.

[7] Ariel Madrigal, Tianyuan Lu, Larisa M Soto, and Hamed S Najafabadi. A unified model for interpretable latent embedding of multi-sample, multi-condition single-cell data. *bioRxiv*, 2023. doi: 10.1101/2023.08.15.553327.

[8] Anna SE Cuomo, Tobias Heinen, Danai Va-

giaki, Danilo Horta, John C Marioni, and Oliver Stegle. CellRegMap: a statistical framework for mapping context-specific regulatory variants using scRNA-seq. *Molecular Systems Biology*, 18(8):e10663, 2022. doi: 10.15252/msb.202110663.

[9] Aparna Nathan, Samira Asgari, Kazuyoshi Ishigaki, Cristian Valencia, Tiffany Amariuta, Yang Luo, Jessica I Beynor, Yuriy Baglaenko, Sara Suliman, Alkes L Price, et al. Single-cell eQTL models reveal dynamic T cell state dependence of disease loci. *Nature*, 606(7912): 120–128, 2022. doi: 10.1038/s41586-022-04713-1.
