# Supplementary material for: Analysis of multi-condition single-cell data with latent embedding multivariate regression
Source: Nat Genet. 2025 Jan 3;57(3):659–67. doi: 10.1038/s41588-024-01996-0 (PMC11906359; doi:10.1038/s41588-024-01996-0)
Supplement: Supplementary file 2 — Reporting Summary [file 41588_2024_1996_MOESM2_ESM.pdf]

nature portfolio

Corresponding author(s): Wolfgang Huber

Last updated by author(s): Aug 1, 2024

Reporting Summary

Nature Portfolio wishes to improve the reproducibility of the work that we publish. This form provides structure for consistency and transparency in reporting. For further information on Nature Portfolio policies, see our Editorial Policies and the Editorial Policy Checklist.

Statistics

For all statistical analyses, confirm that the following items are present in the figure legend, table legend, main text, or Methods section.

n/a	Confirmed	
☐	☒	The exact sample size (*n*) for each experimental group/condition, given as a discrete number and unit of measurement
☐	☒	A statement on whether measurements were taken from distinct samples or whether the same sample was measured repeatedly
☐	☒	The statistical test(s) used AND whether they are one- or two-sided<br>*Only common tests should be described solely by name; describe more complex techniques in the Methods section.*
☐	☒	A description of all covariates tested
☐	☒	A description of any assumptions or corrections, such as tests of normality and adjustment for multiple comparisons
☐	☒	A full description of the statistical parameters including central tendency (e.g. means) or other basic estimates (e.g. regression coefficient) AND variation (e.g. standard deviation) or associated estimates of uncertainty (e.g. confidence intervals)
☐	☒	For null hypothesis testing, the test statistic (e.g. *F*, *t*, *r*) with confidence intervals, effect sizes, degrees of freedom and *P* value noted<br>*Give P values as exact values whenever suitable.*
☒	☐	For Bayesian analysis, information on the choice of priors and Markov chain Monte Carlo settings
☒	☐	For hierarchical and complex designs, identification of the appropriate level for tests and full reporting of outcomes
☒	☐	Estimates of effect sizes (e.g. Cohen's *d*, Pearson's *r*), indicating how they were calculated

Our web collection on statistics for biologists contains articles on many of the points above.

Software and code

Policy information about availability of computer code

Data collection	No software was used for data collection
Data analysis	The complete code necessary to reproduce the results and analysis is available on https://github.com/const-ae/lemur-Paper (permanently stored with Zenodo (https://zenodo.org/doi/10.5281/zenodo.12726369)).<br><br>The software implementation of the proposed new method, LEMUR, is available open source, easy-to-install and well-documented at https://www.bioconductor.org/packages/lemur/ (doi: 10.18129/B9.bioc.lemur) as an R package and on https://pypi.org/project/pyLemur as a Python package.<br><br>We used LEMUR version 1.1.5, scVI version 1.1.2, CPA version 0.8.3, Harmony version 1.1.0, and miloDE version 0.0.9000 (hash: 8803302d). The version of all additional software dependencies can be found in the benchmark/renv.lock and benchmark/conda_env_info files.

For manuscripts utilizing custom algorithms or software that are central to the research but not yet described in published literature, software must be made available to editors and reviewers. We strongly encourage code deposition in a community repository (e.g. GitHub). See the Nature Portfolio guidelines for submitting code & software for further information.

Data

Policy information about availability of data

All manuscripts must include a data availability statement. This statement should provide the following information, where applicable:

- Accession codes, unique identifiers, or web links for publicly available datasets
- A description of any restrictions on data availability
- For clinical datasets or third party data, please ensure that the statement adheres to our policy

All datasets used in this manuscript are publicly available.

Citation	Accession ID
Angelidis (2019)	GSE124872
Aztekin (2019)	bioc::scRNAseq
Bunis (2021)	bioc::scRNAseq
Goldfarbmuren (2020)	GSE134174
Hrvatin (2018)	GSE102827
Jakel (2019)	GSE118257
Sathyamurthy (2018)	GSE103892
Kang (2018)	Zenodo 4473025
Bhattacherjee (2019)	Zenodo 4473025
Skinnider (2021)	Zenodo 4473025
Cano (2020)	Zenodo 5048449
Reyfman (2019)	Zenodo 5048449
Pijuan (2019)	bioc::MouseGastrulationData
Zhao (2021)	GSE148842
Saunders (2023)	GSE202639
Cable (2022)	SCP1663

Research involving human participants, their data, or biological material

Policy information about studies with human participants or human data. See also policy information about sex, gender (identity/presentation), and sexual orientation and race, ethnicity and racism.

Reporting on sex and gender	We did not consider effects of sex or gender in the analysis of the glioblastoma data. There were too few individuals to draw any conclusions. Also, we have no reason to think that such effects, if they existed, would be relevant to the analysis we present.
Reporting on race, ethnicity, or other socially relevant groupings	N/A
Population characteristics	N/A
Recruitment	N/A
Ethics oversight	N/A

Note that full information on the approval of the study protocol must also be provided in the manuscript.

Field-specific reporting

Please select the one below that is the best fit for your research. If you are not sure, read the appropriate sections before making your selection.

☒ Life sciences ☐ Behavioural & social sciences ☐ Ecological, evolutionary & environmental sciences

For a reference copy of the document with all sections, see nature.com/documents/nr-reporting-summary-flat.pdf

Life sciences study design

All studies must disclose on these points even when the disclosure is negative.

Sample size	We chose publicy available datasets whose experimental designs, incl. sample size, were appropriate as a test case for the proposed method / underlying scientific question..
Data exclusions	Across analyses, standard quality control filters were applied to set aside poor quality cells from the single-cell analysis.
Replication	We make all code available to make it easy to reproduce our analysis by third parties. We did not independently replicate the analysis ourselves.

Randomization	Not applicable. We present a complete combinatorial matrix of benchmarks of multiple computational methods each applied to multiple datasets and thus could observe each software in all conditions and did not need to randomize software to condition assignment.
Blinding	The analysts were not blinded while evaluating the benchmark.

Reporting for specific materials, systems and methods

We require information from authors about some types of materials, experimental systems and methods used in many studies. Here, indicate whether each material, system or method listed is relevant to your study. If you are not sure if a list item applies to your research, read the appropriate section before selecting a response.

Materials & experimental systems

n/a	Involved in the study
☒	☐ Antibodies
☒	☐ Eukaryotic cell lines
☒	☐ Palaeontology and archaeology
☒	☐ Animals and other organisms
☒	☐ Clinical data
☒	☐ Dual use research of concern
☒	☐ Plants

Methods

n/a	Involved in the study
☒	☐ ChIP-seq
☒	☐ Flow cytometry
☒	☐ MRI-based neuroimaging

Plants

Seed stocks	N/A
Novel plant genotypes	N/A
Authentication	N/A
